# Elevated Anxiety and Impaired Attention in Super-Smeller, Kv1.3 Knockout Mice

**DOI:** 10.3389/fnbeh.2018.00049

**Published:** 2018-03-19

**Authors:** Zhenbo Huang, Carlie A. Hoffman, Brandon M. Chelette, Nicolas Thiebaud, Debra A. Fadool

**Affiliations:** ^1^Department of Biological Science, Florida State University, Tallahassee, FL, United States; ^2^Program in Neuroscience, Florida State University, Tallahassee, FL, United States; ^3^Institute of Molecular Biophysics, Florida State University, Tallahassee, FL, United States

**Keywords:** olfaction, anxiety, voltage-gated potassium ion channel, Kv1.3, attention deficit, methylphenidate

## Abstract

It has long been recognized that olfaction and emotion are linked. While chemosensory research using both human and rodent models have indicated a change in emotion can contribute to olfactory dysfunction, there are few studies addressing the contribution of olfaction to a modulation in emotion. In mice, olfactory deficits have been linked with heightened anxiety levels, suggesting that there could be an inverse relationship between olfaction and anxiety. Furthermore, increased anxiety is often co-morbid with psychiatric conditions such as attention disorders. Our study aimed to investigate the roles of olfaction in modulating anxiety. Voltage-gated potassium ion channel Kv1.3 knockout mice (Kv1.3−/−), which have heightened olfaction, and wild-type (WT) mice were examined for anxiety-like behaviors using marble burying (MB), light-dark box (LDB) and elevated-plus maze (EPM) tests. Because Kv1.3−/− mice have increased locomotor activity, inattentive and hyperactive behaviors were quantified for both genotypes. Kv1.3−/− mice showed increased anxiety levels compared to their WT counterparts and administration of methylphenidate (MPH) via oral gavage alleviated their increased anxiety. Object-based attention testing indicated young and older Kv1.3−/− mice had attention deficits and treatment with MPH also ameliorated this condition. Locomotor testing through use of a metabolic chamber indicated that Kv1.3−/− mice were not significantly hyperactive and MPH treatment failed to modify this activity. Our data suggest that heightened olfaction does not necessarily lead to decreased anxiety levels, and that Kv1.3−/− mice may have behaviors associated with inattentiveness.

## Introduction

Olfaction is phylogenetically the most ancient sense and has a close association with emotion; it has been well studied that the perception of an odor cue can be altered depending upon reward, threat, or homeostatic state (Hamann, 2003; Krusemark et al., 2013; Nunez-Parra et al., 2014). Unlike other senses, the olfactory system has extensive reciprocal connections with primary emotion areas in the brain such as the amygdala, the hippocampus and the orbitofrontal cortex (Astic et al., 1993; Carmichael et al., 1994; Haberly, 2001). Not surprisingly, a modification in olfactory function may affect emotion. While olfactory function can be disturbed via disease or nutritional state (Rugarli, 1999; Aimé et al., 2007; Thiebaud et al., 2014), olfactory function can also be altered experimentally through surgical (Meredith et al., 1983) or chemical lesions (Slotnick et al., 2007; DiBenedictis et al., 2014), or via genetic engineering (Fadool et al., 2004; Glinka et al., 2012). Interestingly, surgical removal of the olfactory bulb (OB) is classically known to result in anxiety- and depression-like behaviors (for review see Brunjes, 1992). In fact, bulbectomized rodents show behavioral changes that simulate many of those seen in patients with major depression and most of these behaviors can be reversed by chronic antidepressant treatment (Song and Leonard, 2005; Roche et al., 2012; Amigó et al., 2016). As a result, olfactory bulbectomy models have frequently been used to screen for antidepressant drugs (Cairncross et al., 1977; Kelly et al., 1997; Song and Leonard, 2005).

Genetic engineering can also be used to produce either a loss or an accentuation of olfactory ability. For example, Glinka et al. (2012) showed that *Cnga*-2-null mice that lack the cyclic nucleotide-gated ion channel mediating transduction of olfactory signals via the main olfactory epithelium are anosmic, and possess increased anxiety levels compared to mice of normal olfactory ability (Zhang and Firestein, 2002). The authors suggest that anosmia might lead to chronic stress as indicated by measurable elevations in plasma corticosterone levels. Fadool et al. (2004) showed that mice containing a deletion of a predominant voltage-gated potassium channel expressed in mitral cells of the OB (*Kv*1.3−/− mice) have a “Super-smeller” phenotype (Fadool et al., 2004). These mice have heightened olfactory function as measured by olfactory threshold and odor discrimination. If olfactory function and anxiety level are inversely related, we hypothesized that mice with an enhanced olfactory ability might evoke an anxiolytic state or reduction in anxiety. This would be congruent with our previous findings that insulin-induced phosphorylation of Kv1.3 (Fadool et al., 2000, 2011; Marks et al., 2009) and resultant decrease in channel open probability (Fadool et al., 2000) evokes a reduction in anxiety behaviors when intranasally administered to the OB (Marks et al., 2009). Whether the Kv1.3 ion channel could be a pharmacological target to reduce anxiety is also an intriguing possibility.

Increased anxiety is often co-morbid with other psychiatric conditions such impulsivity, inattention and hyperactivity (Biederman et al., 2013; Yüce et al., 2013; Piñeiro-Dieguez et al., 2014). In phenotyping Kv1.3−/− mice for olfactory-related behaviors, we qualitatively observed hyperactivity-like behaviors and quantified increased locomotor activity during the dark cycle as well as irregular ingestive and metabolic activities (Fadool et al., 2004; Tucker et al., 2010). We and others described a thin body type and resistance to diet-induced obesity in the Kv1.3−/− mice that was linked to the conductance of the channel (Xu et al., 2003; Fadool et al., 2004, 2011). Thus, we became curious as to whether olfaction, metabolism and anxiety were interrelated (Palouzier-Paulignan et al., 2012; Krusemark et al., 2013; Kovach et al., 2016).

Ion channels are key actors in the brain and direct modulators of neuronal activity. Targeted deletion of ion channels can have strong behavioral phenotypic changes including attention. For example, deficiency in the sodium channel Na_V_1.1 results in attention disorders causing autistic-like behaviors in mice. More specifically, loss of Na_V_1.1 causes Dravet’s syndrome, a neuropsychiatric disorder that results in intractable seizures, poor social interactions, aversion to social or novel odorants and hyperactivity (Han et al., 2012). In general, inattentiveness and impulsiveness (Taylor, 1998; Sagvolden et al., 2005) has a complex symptomatology. Because individuals with attention disorders have been found to have increased olfactory detection thresholds, sensitivity to bitter stimulants and improved odor sensitivity without a change in identification (Romanos et al., 2008; Weiland et al., 2011; Fuermaier et al., 2018), we hypothesized that loss of the ion channel Kv1.3 may cause an attention phenotype that could be ameliorated with drug treatment. The present study thereby characterized the inattentiveness observed in the Kv1.3−/− mice and determined whether it could be reversed through treatment with methylphenidate (MPH). In parallel, we investigated the interrelationship of anxiety with that of olfactory ability to interrogate whether accentuation of olfactory acuity can reduce anxiety behaviors.

## Materials and Methods

### Animals

All animal experiments were approved by the Florida State University (FSU) Institutional Animal Care and Use Committee (IACUC) under protocol #1427 and were conducted in accordance with the American Veterinary Medicine Association (AVMA) and the National Institutes of Health (NIH Publications No. 8023, revised 1978). For tissue collection, mice were anesthetized with isoflurane (Aerrane; Baxter, Deerfield, IL, USA) using the IACUC-approved drop method and were then sacrificed by decapitation (AVMA Guidelines on Euthanasia, June 2007). Use of the ARRIVE guidelines for reporting animal research was followed in the design of the manuscript (Kilkenny et al., 2010).

All mice (wild-type (WT) C57BL/6J background strain, The Jackson Laboratory, Bar Harbor, ME, USA) were singly-housed in conventional style open cages at the FSU vivarium in accordance with institutional requirements for animal care. Mice were individually housed because group housing has been shown to elevate brain-derived neurotrophic factor (Chourbaji et al., 2008) that phosphorylates Kv1.3 and modifies its biophysical properties (Tucker and Fadool, 2002). Secondarily, group housing in microisolator style cages increases olfactory-based aggression in male mice and results in altered anatomical projections from the main olfactory epithelium to defined synaptic foci in the OB (Oliva et al., 2010; Todrank et al., 2011), thereby altering transmission of olfactory information. To reduce any potential alteration in basal anxiety behavior in our colony, we used two sources of enrichment, namely houses and nestlets. Mice were maintained on a standard control diet (Purina 5001, New Brunswick, NJ, USA). Mice had access to food and water *ad libitum* and experienced a standard 12/12-h light/dark cycle with 7:00 AM lights and 7:00 PM lights off. Behavioral testing was performed 2 h prior to the dark cycle. Both male and female mice were examined for anxiety phenotyping. Because sex-related differences were largely unobserved in the anxiety tests, subsequent experiments only utilized male mice. Kv1.3−/− mice were generated by excision of the Kv1.3 promoter region and one third of the 5′ coding region of C57BL6/J mice (Xu et al., 2003). In accordance with institutional and National Institutes of Health (NIH) guidelines, cages were cleaned weekly and rooms housing mice were examined daily for suitable living conditions. The majority of mice used in this study were 2–5 months of age. For attention and locomotor testing experiments, young mice were defined as 2–5 months of age and older mice were defined as 8–12 months of age.

### Anxiety Testing

#### Marble-Burying Test

**Figure 1:**
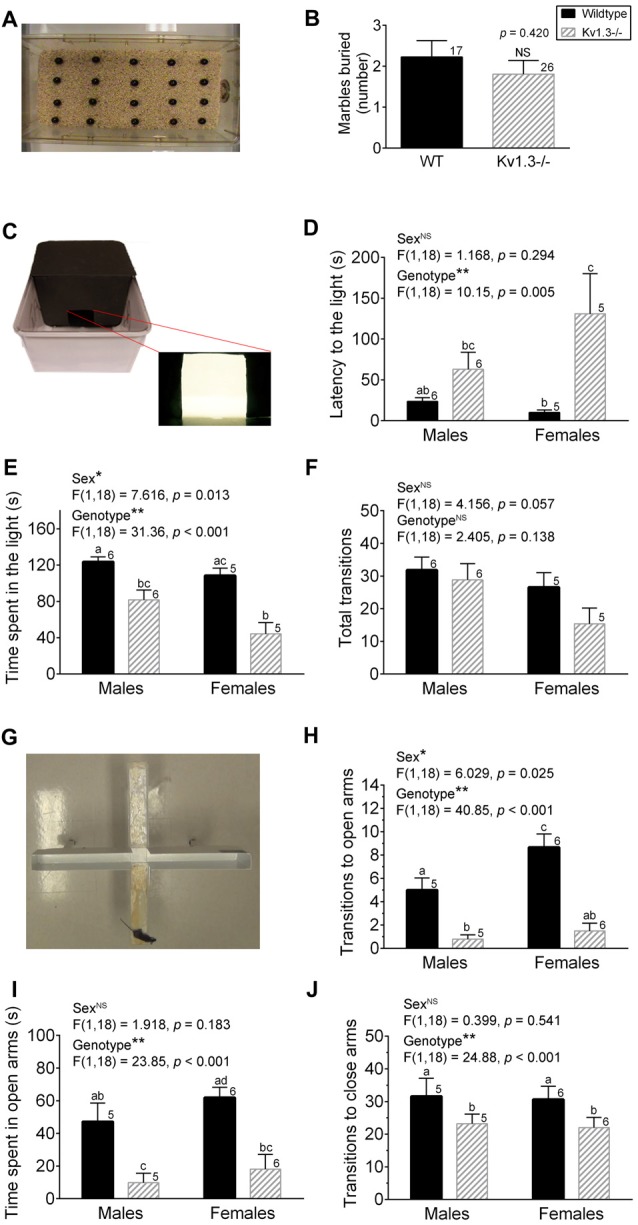
Kv1.3−/− mice exhibit increased anxiety in the light-dark box (LDB) and elevated-plus maze (EPM) apparatus. **(A)** Photograph of the buried marble (MB) test apparatus. **(B)** Bar graph of the number of marbles buried by male wild-type (WT) vs. Kv1.3−/− (Kv1.3−/−) mice. Student’s *t*-test. **(C)** Photograph of the LDB test apparatus. **(D–F)** Bar graphs of the latency to movement to the light box **(D)**, the time spent in the light box **(E)** and the number of transitions between boxes **(F)** comparing WT vs. Kv1.3−/− mice. Two-way analysis of variance (ANOVA), Tukey’s *post hoc* test. **(G)** Photograph of the EPM test apparatus. **(H–J)** Bar graphs of the number of transitions to the open arms **(H)**, the time spent in the open arms **(I)**, the number of transitions to closed arms **(J)** comparing WT vs. Kv1.3−/− mice. Two-way ANOVA, Tukey’s *post hoc* test. **(B,D–F,H–J)** Significantly-different means, *p < 0.05, **p < 0.001. NS = not-significantly different. Different lower case letters indicate significantly-different means in the Tukey’s *post hoc* analysis with genotype and sex as factors. Bars with similar lower case letters indicate means that are not significantly different in the *post hoc* analysis. Number of mice per treatment group as indicated. Data represent mean plus or minus the standard error of the mean (SEM).

Based on the procedure described in Marks et al. (2009), the marble burying (MB) test involved taking mice from their home cage and allowing them to acclimate in an empty rat cage (45 cm [L] × 23 cm [W] × 20 cm [H]) filled with 5 cm of bedding for 15 min. The cage was kept in a small, dark room (1–5 lux) and was covered with a wire lid for the duration of the MB experiment. The mice also did not have access to food or water during testing. After the acclimation period, the mice were returned to their home cage while an evenly-spaced grid of black marbles possessing a light metallic sheen was arranged in the testing cage (Figure 1A). The mice were then placed into the center of the grid of marbles and were free to move around the cage for a 30-min period. After the testing period, the mice were removed and returned to their home cages while the number of buried marbles was counted. Buried marbles were defined as being at least 2/3 covered by bedding. Marbles were never reused between individual animals prior to washing in soap/water and then rinsing in 70% ethanol followed by air drying.

#### Light-Dark Box Test

As described in Marks et al. (2009), the light-dark box (LDB) was constructed from a rat cage (45 cm [L] × 23 cm [W] × 20 cm [H]) that was painted half white and half black. A black lid and removable divider were constructed out of hard, black cardboard. The black divider was positioned at the intersection between the white and black regions to create separate dark (30 lux) and light (595 lux) boxes. The divider also contained a square opening (7 cm [L] × 7 cm [W]) at its base to enable the mouse to easily enter and exit the dark and light boxes. The black lid was placed over the black-painted portion of the box, while a 60-watt light bulb was suspended and positioned over the white-painted portion of the box (Figure 1C). Mice were removed from their home cage and placed directly into the dark box of the testing apparatus. The lid was positioned over the dark box and a small square of hard, black cardboard was held over the opening in the divider for the first 5 s of the testing period. After this time, the square was removed and the mouse was free to move between the boxes for a 5 min testing period. The mouse did not have access to food or water for the duration of the experiment. A video camera (Sony Handycam Camcorder; San Diego, CA, USA) was suspended from a ceiling tile and used to record each testing period. Once the testing period had concluded, the mouse was removed from the testing apparatus and returned to its home cage. The apparatus was wiped with 70% ethanol between animals. After all recordings were completed, Sony Picture Motion Browser software was utilized to digitize the videos and the amount of time the mouse spent in the dark and light boxes was determined.

#### Elevated-Plus Maze Test

Mice were removed from their home cage and placed directly into the center of an elevated-plus maze (EPM; Columbus Instruments, Columbus, OH, USA) positioned an isolated room with a light level of 270 lux. The maze was elevated 45 cm off the ground and possessed four arms, two of which contained barriers (35 cm [L] × 5 cm [W] × 15 cm [H]), while two were open (35 cm [L] × 6.5 cm [W]; Marks et al., 2009; Figure 1G). The mouse was placed at the intersection of the closed and open arms and was able to move freely between the arms for a 5 min testing period. The mouse did not have access to food or water for the duration of the experiment. Following testing, the apparatus was wiped with 70% ethanol between animals. The video camera and the software described above were used to determine the amount of time the mouse spent in the open arms, closed arms and the intersection point.

### Behavioral Attention Testing

#### Oral Gavage

For drug administration, mice were given MPH hydrochloride (MPH, Sigma-Aldrich, St. Louis, MO, USA) or a phosphate buffered saline (PBS) vehicle via oral gavage 60 min prior to testing. The dose of MPH (0.75 mg/kg) was used as described previously (Balcioglu et al., 2009). A dose of 0.75 mg/kg in mice appears to be therapeutically relevant given that this oral dose achieves plasma levels within 15 min as that obtained as a valid occupancy level of the dopamine (DA) transporter in patients (is comparable to 6–10 ng/ml; Balcioglu et al., 2009). Also in accordance with Balcioglu et al. (2009), we used a blunt metal feeding needle (Fisher Scientific, catalog #1020887; Pittsburg, PA, USA) that was lubricated with water prior to insertion of the needle through the mouth and into the stomach of the mouse. MPH or saline was then administered directly into the stomach via the feeding needle and the gavage volume was kept constant at 10 μL/g body weight.

#### Object-Based Attention Test

According to the procedures detailed by Alkam et al. (2011) and Ishisaka et al. (2012), an opaque, plexiglas black chamber was manufactured for use in the object-based attention test. The apparatus consisted of two chambers separated by a removable, sliding divider (acquisition chamber: 40 cm [L] × 40 cm [W] × 22 cm [H]; retention chamber: 40 cm [L] × 20 cm [W] × 22 cm [H]), and the floors of the chambers were covered with a thin layer of standard bedding (Figure 3A). The mice were administered MPH or saline vehicle via oral gavage 30 min prior to beginning the acclimation period. The mouse was then removed from its home cage and placed into the empty, full testing apparatus for 10 min (Figure 3B). The sliding divider was removed during this time to allow the mouse access to both the acquisition (large) and retention (small) chambers. The sliding divider was then reinserted and the mouse was free to explore the large acquisition chamber for 10 min. The sliding divider was finally raised and, using gentle guidance, the mouse was moved into the small retention chamber and was allowed to explore only this area for 10 min. After this final acclimation time, the mouse was removed from the testing apparatus and placed in its home cage while five wooden toys (Imaginarium Wooden Block Set, Wayne, NJ, USA) of various shapes (semi-circle, circle, bridge, rectangle and square) were randomly placed an even distance apart in the acquisition chamber. Two wooden toys, one unique in shape (triangle) and one replicate shape (square) from the five toys placed in the acquisition chamber were also evenly spaced in the retention chamber. The chambers were separated by the sliding divider for the duration of the testing period and the toys used were of similar size, color and smell to prevent undesired bias toward a single shape. The mouse was then placed into the center of the acquisition chamber and was free to explore the five unfamiliar toys for a 3-min timespan. The sliding divider was then raised and the mouse was gently guided into the small retention chamber, where it was free to explore one familiar toy (square) and one unfamiliar toy (triangle) for a 3-min period. The mouse did not have access to food or water for the duration of the experiment. The basis behind this test assumes that if the mouse is able to appropriately divide its attention among the five toys in the acquisition chamber, then it will spend more time attending to the novel toy in the retention chamber. However, if the mouse spends equal or less time on the novel object compared to the familiar object in the retention chamber, then the animal did not properly allocate its attention in the acquisition chamber. All testing was recorded using the Handycam camcorder and associated software as described previously. The time spent on each toy in the acquisition chamber as well as time spent on each toy in the retention chamber were measured from the digitized records. A mouse was considered to be attending to a toy when its body was oriented toward it and it was within one nose-length of the object. The amount of time spent on the unfamiliar toy compared to the amount of time spent on both toys in the retention chamber was defined as the recognition index (Alkam et al., 2011).

**Figure 2:**
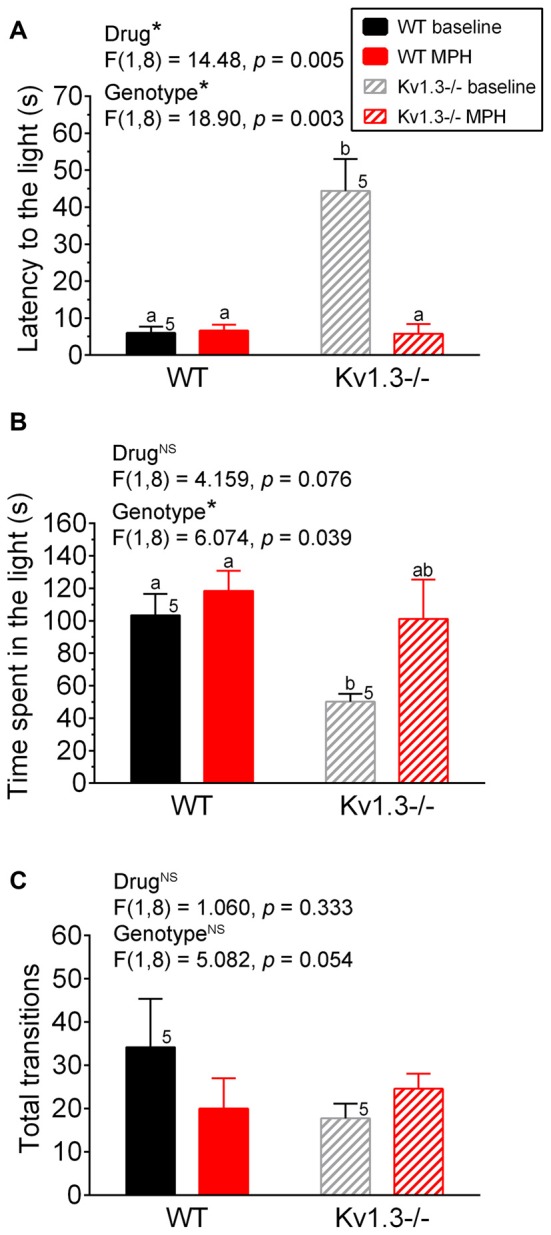
Anxiety disorder in male Kv1.3−/− mice can be alleviated by methylphenidate (MPH) treatment. **(A)** Bar graph of latency of first movement to the light box of the LDB, **(B)** the time spend in the light box of the LDB, or **(C)** the total transitions between the light and dark box of the LDB. In all experiments, WT vs. Kv1.3−/− mice were first treated with saline (baseline) and then with MPH. Two-way repeated measure (RM) ANOVA, Tukey’s *post hoc* analysis with drug and genotype as factors. Same notations as in Figure 1.

**Figure 3:**
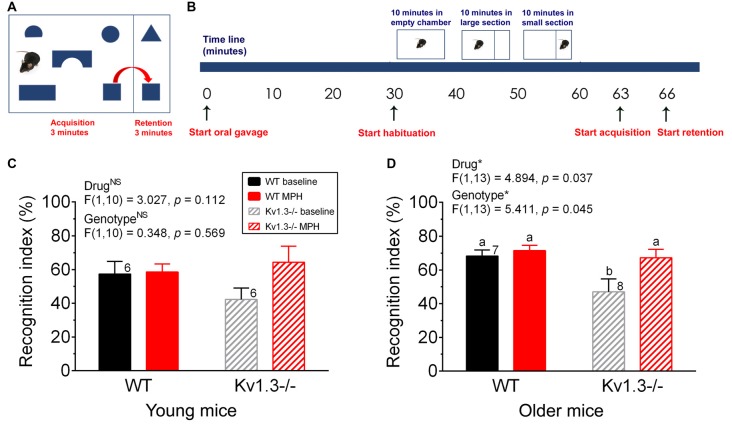
Object-based attentional deficits of male Kv1.3−/− can be ameliorated by MPH treatment. **(A)** Schematic diagram of the object-based attention test apparatus whereby mice are permitted a 3-min interval in the acquisition chamber followed by a 3-min testing interval in the retention chamber. **(B)** Schematic time line of the experiment. Preceding habituation, each mouse was orally gavaged (start oral gavage) with saline or MPH and was returned to its home cage for 30 min. After this period, habituation began and occurred in three stages, each 10 min in duration. For the first 10 min, the mouse was free to explore the entire empty apparatus. A removable divider was then placed within the apparatus, dividing the cage into a large acquisition chamber and a small retention chamber. The mouse was free to explore the large acquisition chamber for 10 min, then the small retention chamber for 10 min. After habituation, the mouse was placed in the center of the acquisition chamber that now included the shape objects and was permitted to roam the chamber for 3 min (the acquisition phase). After this time, the removable divider was raised and the mouse was gently guided into the retention chamber. Once the mouse was in the smaller chamber, the divider was slid back into place and the mouse was permitted to roam the retention chamber for 3 min (the retention phase). Bar graph of the recognition index for **(C)** young vs. **(D)** older mice. **(C,D)** young = 2–5 months, older = 8–12 months of age. Same statistical analyses and notations as in Figure 2.

#### Locomotor Test

**Figure 4:**
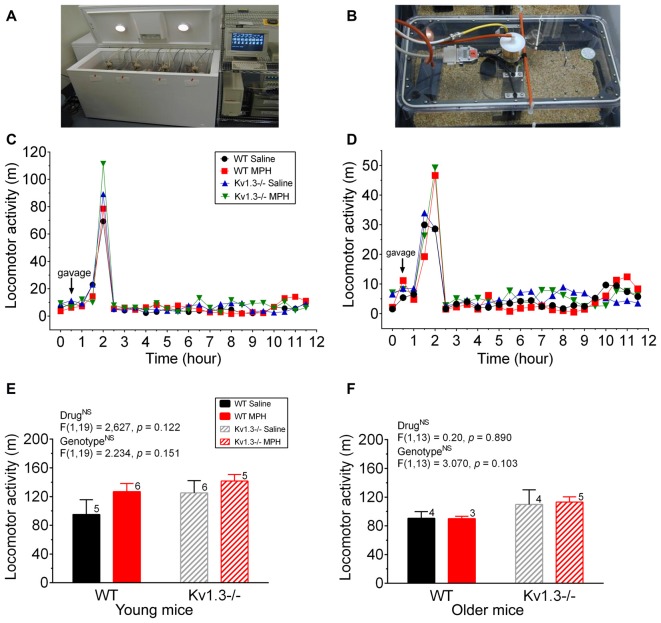
Locomotor activity is not significantly altered by MPH treatment. **(A,B)** Photograph of a custom-made metabolic chamber **(A)** and individual home cage that is sealed to perform indirect calorimetry as well as positioned on a platform to detect movement in a 50-mm unidirectional movement **(B)**. **(C,D)** Line graph of the mean locomotor activity following an oral gavage of saline or MPH (arrow) initiated 2 h into the dark cycle for young **(C)** and older mice **(D)**. **(E,F)** Bar graph of the mean locomotor activity for 10 h following oral gavage for young **(E)** and older mice **(F)**. Same statistical analyses and notations as in Figure 1.

Metabolic chamber analysis involved removing mice from their home cage and placing them into sealed chambers that recorded total locomotor movement and water, food and air consumption over a 24-h period (Williams et al., 2000; Tucker et al., 2008). While in the chambers, the mice had unlimited access to food, water and one enrichment item. The metabolic chambers were built by the FSU machine shop and were derived from standard rat cages (Figure 4A). Eight cages were stored in two recycled deep freezers to enable the air levels and temperature of the cages to be controlled. A computer program was used to record the pressure, oxygen and carbon dioxide levels as well as the temperature of the chambers and the total distance moved by each mouse over the 24-h day. Only data from the dark cycle were analyzed for these experiments. Each day at 6:00 PM, the mice were weighed and the food and water from each chamber were removed and weighed to determine how much the mice had consumed over the previous 24-h period. The food and water were then disposed of and fresh equivalents were weighed and presented to the mice. The food utilized was standard mouse chow (Purina 5001) that had been ground into a fine powder. The mice were kept in the chambers for a minimum of 6 days total and experienced a standard 12/12-h light/dark cycle during this time from 7:00 AM light on to 7:00 PM light off. The cages were not changed or cleaned for the duration of the 6-day acclimation, but were only cleaned before a new mouse was placed in the chamber. After acclimation, mice were weighed at 6:00 PM and gavaged with either saline or MPH at 9:00 PM. There was a 2 h time lapse between initiating the dark cycle (7:00 PM) and initiating gavaging (9:00 PM) to enable the mice time to acclimate to the dark cycle before beginning testing. After gavaging, the total distance traveled from 9:00 PM to 7:00 AM (10 h block) was recorded. The total distance traveled per mouse for each of the three nights of testing was averaged to yield one mean distance value per mouse.

#### Tissue Harvest for HPLC and SDS PAGE/Western Analyses

Mice were anesthetized with isoflurane (Aerrane; Baxter, Deerfield, IL, USA) using the IACUC-approved drop method and were then sacrificed by decapitation (AVMA Guidelines on Euthanasia, June 2007). OBs were quickly harvested after decapitation. Tissues were immediately frozen in dry ice/100% ethanol. The HPLC measurements of DA and 3,4-dihydroxyphenylacetic acid (DOPAC) were performed by the Vanderbilt Neurochemistry core at Vanderbilt University (Nashville, TN, USA). For SDS PAGE analysis, OBs were homogenized in NP40 PPI for fifty strokes with a Kontes tissue grinder (size 20) on ice, and cytosolic proteins were extracted as previously described (Tucker and Fadool, 2002). The cytosolic proteins (25 μg/lane) were separated on 8%–10% acrylamide gels by SDS-PAGE and electrotransferred to nitrocellulose blots. Nitrocellulose was incubated overnight at 4°C with the primary antibodies: anti-tyrosine hydroxylase (TH), anti-DA receptor type II (D2DR) and extracellular signal-related kinase (ERK). The anti-TH (applied at 1:500 dilution) was acquired from Cell Signaling Technology (Danvers, MA, USA, cat# 2792) and is a polyclonal antibody produced by injecting rabbits with a synthetic peptide that corresponds to the amino-terminal sequence of human TH. The anti-D2DR (applied at 1:500) is from Millipore (Temecula, CA, USA, cat# AB5084P) and is a polyclonal antibody produced by injecting rabbits with a 28 amino acid peptide sequence within cytoplasmic loop #3 of the human D2 receptor. The anti-D2DR recognizes both the long and short form of the receptor. The anti-ERK (applied at 1:2000) was acquired from Cell Signaling Technology (cat# 9102) and is a polyclonal antibody produced by injecting rabbits with a synthetic peptide derivative of the C-terminus of rat ERK1. The anti-ERK recognizes both ERK1 and ERK2. Enhanced chemiluminescence (ECL; Amersham-Pharmacia) exposure on Fugi Rx film (Fisher) was used to visualize labeled proteins. The film autoradiographs were analyzed by quantitative densitometry using a Hewlett-Packard Photo Smart Scanner (model 106–816, Hewlett Packard, San Diego, CA, USA) in conjunction with Quantiscan software (Biosoft, Cambridge, England). Immunodensity ratios (Kv1.3−/− over WT) were calculated, normalized and analyzed as described previously (Tucker and Fadool, 2002).

### Statistical Analyses

GraphPad Prism 6.0 (GraphPad Software, Inc., La Jolla, CA, USA) was utilized for statistical analyses. For each analysis, data were first confirmed to be normally distributed using the D’Agostino and Pearson omnibus normality test. For anxiety tests comparing genotyping differences, a two-way randomized analysis of variance (ANOVA) was applied with sex and genotype as factors. For anxiety, attention and locomotor tests involving MPH treatment, a two-way, repeated measures (RMs) ANOVA was applied using drug and genotype as factors. *Post hoc* tests for multiple comparisons were performed with a Tukey’s correction. Student’s *t*-test and non-parametric Mann-Whitney test were applied for two-sample population studies for the HPLC and SDS PAGE experiments. Statistical significance was determined at the 95% confidence level (α ≤ 0.05) unless otherwise noted. Values are reported as the mean ± standard error of the mean (SEM). Data were graphed using Origin 8.0 (MicroCal Software, Northampton, MA, USA) or Photoshop CS7 (Adobe, San Jose, CA, USA). In the *post hoc* analyses for ANOVA tests, bar graphs with different lower case letters indicate significantly-different means. Those with similar lower case letters have means that are not significantly different in the *post hoc* analyses.

## Results

### Kv1.3−/− Mice Exhibit Increased Anxiety in the LDB and EPM Apparatus

We initiated anxiety testing in the Kv1.3−/− mice due to the reported comorbidity of ADHD and anxiety disorders (Biederman et al., 2013; Yüce et al., 2013; Piñeiro-Dieguez et al., 2014). We used three different paradigms to assess anxiety-like behaviors in the Kv1.3−/− mice—the MB test, the LDB test, and the EPM test (Figure 1). Species-typical behaviors such as burrowing and digging have been employed as sensitive measures to screen the effects of suspected anxiolytic drugs but there are still debates in the literature as to whether this test detects anxiety, depression, or obsessive-compulsive behavior in rodent models (Deacon, 2006). Only male mice were available during our MB tests, but the other two tests used mice of both sexes. The number of marbles buried is correlated to an increase in anxiety or related disorder. Using a 30-min testing interval (see “Materials and Methods” section), the number of marbles buried was not significantly different across genotypes (Student’s *t*-test, *p* = 0.420; Figure 1B). In the LDB test, the latency for the Kv1.3−/− mice to make the first move to the light box was significantly delayed (Figure 1D; sex: NS, *p* = 0.294; genotype: *p* = 0.005; interaction: *p* = 0.124) and the total time spent in the light box was significantly reduced compared with that of WT mice (Figure 1E; sex: *p* = 0.013; genotype: *p* < 0.001; interaction: *p* = 0.257) when applying a two-way ANOVA using genotype and sex as factors; Tukey’s *post hoc* test. While these metrics implicated an increased anxiety level in the Kv1.3−/− mice, there was not a significant difference in exploratory behavior as assessed by total number of transitions between boxes (Figure 1F; 2-way ANOVA, sex: *p* = 0.057; genotype: *p* = 0.138; interaction: *p* = 0.382) although there was a trend that the Kv1.3−/− moved less in this assay and particularly for that of females. The third and final anxiety test represented one of threatening or real danger for the mouse given that the apparatus has open arms that are 42 cm off the floor in the EPM. The number of transitions to the open arms of the maze was less than 10% of the total transitions for Kv1.3−/− mice (Figure 1H vs. J; 2-way ANOVA, sex: *p* = 0.025; genotype: *p* < 0.001; interaction: *p* = 0.113) that spent a significantly shorter duration of their testing time in the open arms (Figure 1I; 2-way ANOVA, sex: *p* = 0.183; genotype *p* < 0.001; interaction: *p* = 0.705). Kv1.3−/− mice had significantly less transitions to closed arms; a test that typically is an indicator of general locomotor activity (Figure 1J; 2-way ANOVA, sex: *p* = 0.541; genotype: *p* < 0.001; interaction: *p* = 0.939). Because Kv1.3−/− mice have been reported to have more activity than that of WT mice when in their home cage without stressors (Fadool et al., 2004), we judge it is unlikely they have an impaired locomotor ability in non-threatening environments. In anxiety prone environments, however, we observed that approximately 20% of the Kv1.3−/− mice exhibited a freezing-like behavior in the open arms of the EPM. If the animals immobilized in the open arms for over 15 s, the data were excluded and recorded as a freezing event.

### MPH Treatment Ameliorates Kv1.3 Anxiety and Attention Deficit

Next we examined whether the significant genotypic difference we observed for the anxiety-like behavior in the LDB could be alleviated for mice following MPH treatment. First, both WT and Kv1.3−/− male mice were administered saline via oral gavage to determine their basal anxiety levels. As shown in Figure 2, Kv1.3−/− mice showed increased latency to the light box (Figure 2A), and spent less time in the light box (Figure 2B), which indicated increased anxiety, similar to what we demonstrated previously in the absence of oral gavage (Figure 1). After 4 days, the same mice were administered MPH by oral gavage, and anxiety levels were retested. As shown in Figure 2B, MPH treatment alleviated the anxiety of the Kv1.3−/− that now spent time in the light box, which was not significantly different than that of WT mice (two-way repeated ANOVA, drug* p* = 0.076, genotype* p* = 0.039; interaction: *p* = 0.298). The most marked effect was the first latency to the light that was 8× as long in the Kv1.3−/− vs. WT mice prior to MPH treatment, which after MPH treatment, fell to less than 5 s and was similar to latency times recorded for WT mice (Figure 2A; two-way repeated ANOVA, drug* p* = 0.005, genotype* p* = 0.003; interaction: *p* = 0.004). In order to rule out that decreased latency to the light could be caused by MPH administration enhancement of locomotor activity, we examined total transition time between the light and dark box (Figure 2C). There was no significant effect of MPH on locomotor activity (Two-way repeated ANOVA, drug: *p* = 0.333; genotype: *p* = 0.054; interaction: *p* = 0.019).

Because anxiety is usually co-morbid with many psychiatric disorders, we explored whether Kv1.3−/− mice also exhibited other cognitive defects. Using a relatively new object-based attention testing paradigm we examined the attention levels of young (2–5 months) vs. older (8–12 months) mice (Figure 3A). Both WT and Kv1.3−/− mice were initially administered saline via oral gavage to determine their basal attention levels, again under an oral route of administration. The test animal’s attention was indicated by a calculated recognition index (see “Materials and Methods” section). Mice with better attention abilities will be able to spend less time with the familiar object and a greater amount of time with the unfamiliar object, yielding a higher recognition index. After 2 weeks, the same mice were administered MPH via oral gavage and their recognition index was recomputed. While in the acquisition phase (Figure 3B), the mice spent approximately equal amounts of time exploring each of the five toys (data not shown), thus indicating there was no bias towards a particular shape. Older Kv1.3−/− mice had a significantly reduced recognition index that was elevated to that of WT mice following MPH treatment (Figure 3D; two-way repeated ANOVA, drug:* p* = 0.037; genotype: *p* = 0.045; interaction: *p* = 0.133). This pattern of behavior was also observed for the young mice but did not reach statistical significance (Figure 3C; two-way repeated ANOVA; drug: *p* = 0.112, genotype: *p* = 0.569; interaction: *p* = 0.152).

### Locomotor Testing

In addition to attention deficits, we examined whether MPH treatment could mitigate the increased activity of Kv1.3−/−, which have been reported to be elevated over that of WT mice during the dark cycle (Fadool et al., 2004). Metabolic chamber analysis (Figures 4A,B) was performed on mice of both genotypes to measure locomotor activity, and thus indirectly measure hyperactivity. Mice were gavaged 2 h into the dark cycle. The amount of locomotor activity within the first 60 min following the gavage was recorded as well as the total number of meters moved for the remaining 10 h of the dark cycle. Within the first hour following oral gavage, older mice treated with MPH trended to have an elevated activity over that of saline-treated mice, regardless of genotype (Figure 4C; young mice; two-way ANOVA, drug: *p* = 0.27; genotype: *p* = 0.53; interaction: *p* = 0.86 and Figure 4D; older mice; two-way ANOVA, drug: *p* = 0.15; genotype: *p* = 0.25; interaction: *p* = 0.66). It was also evident that the gavage treatment itself elicited a general increase in locomotor activity because post-gavage activity levels were 5–6× greater than that of pre-gavage activity levels and then decreased after roughly 90 min following administration of saline or drug. In tabulating cumulative activity over the 10 h dark cycle, the young Kv1.3−/− mice trended to have greater locomotor activity compared to that of WT mice, and MPH treatment increased activity for both Kv1.3−/− and WT mice, though there were no statistical significant differences (two-way ANOVA, drug: *p* = 0.122; genotype: *p* = 0.151; interaction: *p* = 0.135; Figure 4E). For older animals, Kv1.3−/− mice also trended towards higher locomotor activity when calculated over the 10 h dark cycle, however, MPH treatment had no effect on activity for either genotype (two-way ANOVA, drug: *p* = 0.890; genotype: *p* = 0.103, interaction: *p* = 0.239; Figure 4F).

### Dopamine Measure and Expression of Down-Stream Signaling Proteins

**Figure 5:**
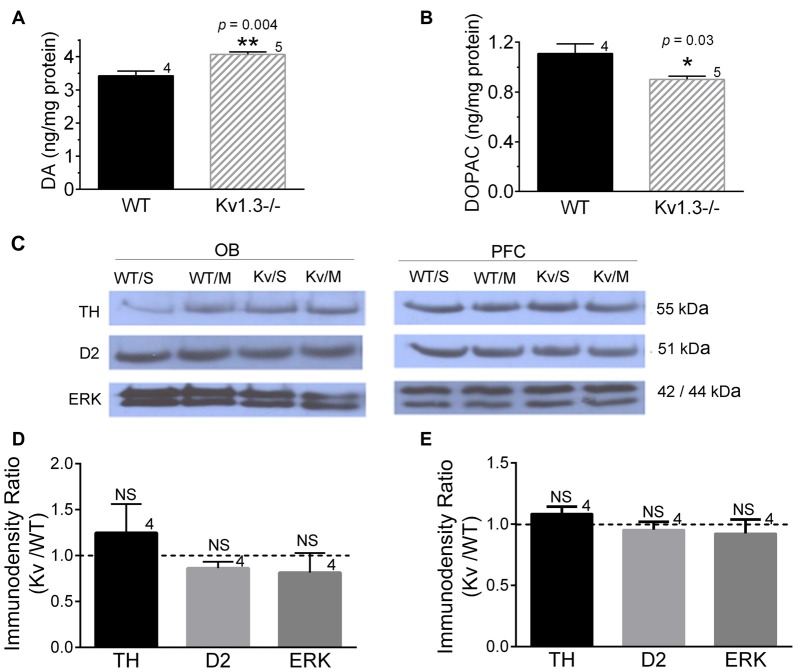
Dopaminergic signaling differences in WT vs. Kv1.3−/− mice. **(A)** Bar graph of the HPLC determination of dopamine (DA) and **(B)** 3,4-dihydroxyphenylacetic acid (DOPAC) in the olfactory bulb (OB). **(C–E)** Western blot analysis **(C)** and associated quantitative densitometry (bottom) for dopaminergic signaling proteins expressed in **(D)** the OB and in **(E)** the prefrontal cortex (PFC). Mice were gavaged with saline control (S) or MPH (M) 30-min prior to tissue collection. Western blot analysis of saline- vs. MPH-treated mice demonstrated no significant changes; therefore analyses were pooled to compare immunodensity ratios across Kv1.3−/− vs. WT mice. Pixel immunodensity values were determined for each protein band and then expressed as a ratio (dashed line = no expression difference, or 1.0). WT/S, WT with saline; WT/M, WT with MPH; Kv/S, Kv1.3−/− with saline; Kv/M, Kv1.3−/− with MPH; TH, tyrosine hydroxylase; D2, DA receptor 2; and ERK, extracellular signal-related kinase. **(A,B)** Student’s *t*-test, *p* < 0.05, **(D,E)** Mann-Whitney test, *p* > 0.05, sample size represents the number of mice. kDa, kilodaltons. Full size SDS PAGE Western blots can be found in the Supplementary Figure S1.

Because dopaminergic signaling may be involved in modulating anxiety-like behaviors (Sullivan et al., 2014), we explored whether there were differences in basal DA or MPH treatment effects on DA signaling in WT vs. Kv1.3−/− mice. An HPLC analysis of the OB of untreated animals demonstrated that Kv1.3−/− mice had significantly higher DA levels, while they contained less of the DA metabolite DOPAC (Figures 5A,B; Student’s *t*-test, *p* < 0.05). Western blot analysis of saline- vs. MPH-treated mice demonstrated no significant changes in TH, DA receptor 2 (D2DR) protein levels, or extracellular-signal-regulated kinase (ERK) within the OB (Figure 5C, left) or the prefrontal cortex (PFC; Figure 5C, right), therefore analyses were pooled to compare immunodensity ratios across Kv1.3−/− vs. WT mice for each protein within (Figure 5D) the OB and (Figure 5E) the PFC (not significantly different, Student’s *t*-test, *p* > 0.05).

## Discussion

By behavioral phenotyping mice with a deletion of the Kv1.3 potassium ion channel, we discovered that the “Super-smeller” mice (Fadool et al., 2004) had an increased level of anxiety that was ameliorated by treatment with MPH. While younger Kv1.3−/− mice trended to exhibit reduced attention to an object-based attention task, older Kv1.3−/− mice performed with a significant deficit, which also was ameliorated by MPH treatment. Because enhanced locomotor activity of Kv1.3−/− mice was unchanged by MPH treatment, this transgenic mouse line might be a favorable model for inattentiveness. Our results suggest that anxiety and olfactory function may not necessarily be inversely related in all instances of change in olfactory perception.

Contrary to our hypothesis, the Kv1.3−/− mice largely exhibited behaviors consistent with an elevated anxiety in two of the three behavioral tests we performed. We initially surmised that the “Super-smeller” phenotype of the Kv1.3−/− mice would predict a reduction in anxiety due to the reported enhancement in stress and anxiety in mouse models of anosmia, such as that of the *Cnga2*-null lines (Glinka et al., 2012). However, given our findings that the Kv1.3−/− mice with an enhanced olfactory ability for both odor discrimination and threshold (Fadool et al., 2004) have increased anxiety, we must conclude that olfactory function and anxiety have a relationship that is not strictly inversely related. We cannot exclude that targeted deletion of Kv1.3 outside the olfactory system could also evoke a change in emotion that is not linked to olfactory perception. Alternatively, a change in olfactory perception, either enhanced or dampened, may reflect an altered or stressed state to increase anxiety. It seems that aberrant olfaction could be associated with anxiety. In fact, in humans, emotion has been linked with altered olfaction in both directions. It has been shown that a negative emotional state reduces olfactory sensitivity (Pollatos et al., 2007), while enhanced olfactory sensory perception with anxiety has also been reported (Krusemark and Li, 2012).

Consistent with this idea are another genetically-engineered line of mice in which greater than 95% of all sensory neurons express the same odorant receptor, or the M71 monoclonal nose model (Fleischmann et al., 2008). These mice are not anosmic, although they lack detection of their preferred ligand, acetophenone and they can detect other odorants, despite impairments in associative odor learning tasks and reduced discrimination. Nonetheless monoclonal nose mice have increased stress and heightened anxiety (Glinka et al., 2012). Because olfaction is the rodent’s primary sensory modality and source of major interaction with its external environment, it is not unexpected that a change in any direction of its olfactory function and processing of odor information could modify behavior to produce stress or anxiety.

Prior to our investigation, few animal studies had been performed concerning the relationship between olfactory function and anxiety. In rodents, it has been demonstrated that associative learning (fear—foot shock) can facilitate olfactory nerve synaptic output to glomerular activity to change the neural representation of what become predictive threat odorants (Kass et al., 2013). In human subjects, Krusemark et al. (2013) interestingly were able to drive changes in anxiety levels to the extent that previously neutral or innocuous odorants became unpleasant and took longer to detect. Using fMRI they were able to discern that these now aversive odorants induced augmented responses in higher olfactory cortices and the anterior cingulate cortex (Krusemark et al., 2013). Our mouse studies utilized three different types of anxiety paradigms including MB, LDB and EPM. MB and LDB tests examine basal level of anxiety in non-threatening environments, whereas the EPM introduces a threatening or actual danger of being 42 cm off the ground. The MB task has been demonstrated to be an effective assessment of anxiety (Borsini et al., 2002) but is also utilized to measure the degree of repetitive compulsivity or obsessive-compulsive disorder (OCD), for example, when transgenic mice are devoid of producing serotonin (Angoa-Pérez et al., 2013). Alternatively, supplementing with serotonin-active compounds or a variety of anxiolytics that act to reduce anxiety, depression, or OCD, will oppositely reduce burying or digging behaviors (Borsini et al., 2002; Deacon, 2006). Because Kv1.3−/− mice did not bury significantly greater number of marbles than that of WT mice, they do not appear to be a model for repetitive compulsivity, although the other two paradigms, LDB and EPM, indicated a significant difference in anxiety-like behaviors, and ones that could be ameliorated by MPH treatment. The LDB can predict basal anxiolytic- or anxiogenic-like activity in mice, whereby transitions have been reported to be an index of activity-exploration over that of time spent in each compartment, which might habituate over time, but is a reflection of aversion (Bourin and Hascoët, 2003). In LDB, mice with targeted deletion of Kv1.3 channel spent less time in the light chamber and had a greater latency to move to the light chamber, indicating anxiogenic behaviors. The level of transitions between chambers trended to be less for female Kv1.3−/− mice that perhaps additionally had reduced activity-exploration behaviors. In the EPM, Kv1.3−/− mice not only spent less time in the open arms, they transitioned to them less frequently and their total transitions between compartments were less independent of sex. In comparison to treatments in which insulin is intranasally delivered to phosphorylate its Kv1.3 substrate, which is known to decrease mean open time of the channel (Fadool et al., 2011), mice with reduced Kv1.3 activity but not targeted deletion have anxiolytic-like behaviors or reduced anxiety in terms of more time in the light chamber of the LDB as well as increased time in open arms of the EPM (Marks et al., 2009). Even though posttranslational modification of the channel and targeted deletion both disrupt channel function, phosphorylation of Kv1.3 channel can serve as a signalplex for a variety of protein-protein interactions (Cook and Fadool, 2002; Marks and Fadool, 2007; Colley et al., 2009; Marks et al., 2009) and may not necessarily correlate to a complete absence of the ion channel protein. Intranasal insulin would also not be expected to have phosphorylation effects on a single substrate.

Studies have begun to examine the effect of MPH on anxiety in control vs. disorder populations. In our investigation of the ability for MPH to mitigate the anxiety behaviors noted in the Kv1.3−/− mice, the time spent in the light chamber of the LDB was significantly reduced in Kv1.3−/− mice and increased compared with that of WT animals following MPH administration. The Kv1.3−/− mice had a delayed latency to enter the light chamber that was 4× as long as that exhibited by WT mice—which again was completely ameliorated following MPH administration to the short 10 s latency observed for that of WT mice. One could alternatively interpret that the reduced latency to the dark in MPH treated Kv1.3−/− mice could be due to the delivery of a potent stimulant drug, rather than reduced anxiety, however, the MPH treated Kv1.3−/− mice spend equal amount of time in the light as WT mice, indicating this was not attributed to increased movement. MPH had no effect on anxiety in the WT cohort. Interestingly, in comparing our data to that of a human subjects study, Segev et al. (2016) also found that MPH was ineffective in reducing anxiety across a control group or elicited mild enhancement in anxiety, but significantly decreased anxiety in individuals with subclinical or state-anxiety (Segev et al., 2016). Golubchik et al. (2017) similarly report changes in anxiety for Asperger/and ADHD co-morbid patients as opposed to unafflicted individuals (Golubchik et al., 2017).

The object-based attention test employed for this study was a relatively new paradigm for testing animal behaviors (Alkam et al., 2011; Ishisaka et al., 2012), but provided an advantage in terms of the absence of training required to perform the task and the reduced amount of time involved in completing the task. We have previously demonstrated that Kv1.3−/− mice exhibit no deficits in short- (1 h) or long-term (24 h) memory when examined in a simple object memory recognition test (Tucker et al., 2012). Therefore the reduced performance of Kv1.3−/−mice in the object-based attention test that was ameliorated by MPH treatment was not likely attributed to any disruption in memory. Although our object-based attention test had the limitation that it could not fully examine ADHD characteristics by utilizing typical multiple fixed-interval/extinction schedules of reinforcement (FI/Ext schedules) to allow sustained attentiveness, impulsivity, or hyperactivity to be measured (Sagvolden et al., 2005), it could potentially be used for large-scale preclinical screening of drug candidates, for example. An operant attention task (5-choice serial reaction time task; Asinof and Paine, 2014) is a more demanding task that could be examined for a refined exploration of the type of inattentiveness defect.

While young Kv1.3−/− mice trended to have a reduced recognition index compared to that of WT mice, older mice exhibited a significantly reduced index that was restored following MPH treatment. It is likely that the attention deficit present in the Kv1.3−/− mice is not strongly age-dependent because the reduced attention deficit observed in the young animals is similarly restored following MPH treatment and may be the result of lesser sample size. The fact that MPH treatment could ameliorate a reduced recognition index in both age groups implicates that Kv1.3−/− mice could be suitable models for inattentiveness or cognition problems. The amount of MPH presented to each mouse was equivalent to the clinical dose given to human subjects diagnosed with ADHD (Balcioglu et al., 2009); however, only a single dose was utilized for this study, while some studies have included several dosages for comparison (Balcioglu et al., 2009; Zhu et al., 2012). A stronger effect on the attention levels of either Kv1.3−/− or WT mice may have been observed with the use of a higher dose or with the use of repeated or chronic administration of MPH.

Our use of a metabolic chamber to measure locomotor activity was an indirect method to assess hyperactivity, nonetheless we found that the Kv1.3−/− mice did not have significantly elevated, sustained hyperactivity levels. The type of rapid locomotor activity we observed by our animals was unexpected and a slower rate of lessened activity over time would be more anticipated. Any handling effect would have been anticipated to be closer to the time of gavage rather than 30–60 min following. Our administered dose of MPH may have elicited a rapid onset of locomotor activity, which was just not sustained. Another interpretation based upon studies of Kuczenski and Segal (2005) is that “clinical doses” of MPH (~0.5 mg/kg) are near or below threshold for the induction of locomotor activation, although they retain the ability to affect extracellular neurotransmitter release (Kuczenski and Segal, 2005; Venkataraman et al., 2017. Thus our dose of 0.75 mg/kg was clinical but perhaps only marginally affected locomotor activity. Interestingly, route of delivery is also a factor. It is known that intraperitoneal administration of MPH causes a higher peak plasma drug level (Patrick et al., 1984) vs. that of oral administration. In fact, Kuczenski and Segal (2001, 2002) found that lower doses of MPH (0.75–3.0 mg/kg) had increased locomotor activity when administered IP but not when provided orally. Previous studies involving metabolic chamber testing have indicated that Kv1.3−/− mice had significantly greater locomotor activity than WT mice (Fadool et al., 2004); whereas here we noted the same trend although it did not reach statistical significance. Additionally, treating young mice with MPH resulted in increased locomotor activity, regardless of genotype. Because MPH is a stimulant, its administration to normally functioning individuals typically results in increased activity levels while its administration to individuals with inattention typically results in reduced activity. Both young WT and Kv1.3−/− mice exhibited a trend toward greater activity with MPH treatment. Whereas possibly due to age factor, older mice lacked locomotor response to MPH treatment. Overall, these results indicate that Kv1.3−/− mice are not a suitable model of hyperactivity.

Stimulants exert their effectiveness by acting on both the dopaminergic (DA) and norepinephrine systems in the brain (Biederman and Faraone, 2005; Hellwig-Brida et al., 2011). Neurodegenerative diseases that have a similar loss of DA, such as Parkinson’s Disease, are also associated with a change in olfactory sensitivity (Mesholam et al., 1998). We therefore focused our protein biochemistry analysis on DA and its downstream metabolites. MPH, in particular, is a DA transporter (DAT) inhibitor, thus resulting in an increase in extracellular DA concentrations through the release of DA from intracellular storage (Carey et al., 1998; Biederman and Faraone, 2005). While we did not explore direct MPH induction of DA, others have found it to increase DA release from the PFC, nucleus acumens and caudate-putamen (Carey et al., 1998) and also found that it increases frontal cortex and striatal activation (Hellwig-Brida et al., 2011). Stress can increase DA release in the PFC (Deutch and Roth, 1990; Finlay et al., 1995) and elevated D2DR availability in the orbitofrontal cortex has been linked in humans with social anxiety disorder symptoms (Plavén-Sigray et al., 2017).

We found that Kv1.3−/− mice have an elevated, basal level of DA in the OB and a reduction in one of its metabolites, DOPAC. While we don’t know its comparative level in the PFC, the level of TH, ERK and D2DR were all unchanged in the PFC, suggesting that dopaminergic signaling in this region is likely not responsible for anxiety-like behaviors of the Kv1.3−/− mice. While vesicular release of DA is typically cleared by DAT in most brain regions, the OB predominantly uses catechol-O-methyl-transferase (COMT) over that of DAT (el-Etri et al., 1992; Cockerham et al., 2016), so it is not inconceivable that MPH would have no real targeted effect in the OB. The fact that DA is elevated in the OB of Kv1.3−/− mice, however, might suggest a differential degree of dopaminergic modulation in the Kv1.3−/− mice, leading to a change in olfactory sensitivity. For example, olfactory gain control driven by local juxtaglomerular interneurons via traditional lateral inhibition is regulated by co-release of DA and GABA from deep short axon cells to change firing frequency of the major output neurons, namely mitral/tufted cells (Vaaga et al., 2017).

In order for an animal model to be similar to a human disease in terms of etiology, it must mimic the behavioral characteristics of the disorder (Davids et al., 2003; Sagvolden et al., 2005). While Kv1.3−/− mice are suitable models of inattentiveness, deletion of the Kv1.3 channel in humans has not, as of yet, been linked to ADHD-type behaviors. In future studies, Kv1.3−/− mice may afford utility in exploring attentional problems associated with anxiety or depression.

## Author Contributions

ZH was responsible for data collection of anxiety tests and DA measure, analysis of the data and preparing the first draft of the manuscript. CAH collected attention test and locomotor test data, analysis of the data, and preparing the first draft of the manuscript. BMC performed the Western blot experiments. NT and DAF contributed to experiment ideas and design, and DAF wrote the final manuscript.

## Conflict of Interest Statement

The authors declare that the research was conducted in the absence of any commercial or financial relationships that could be construed as a potential conflict of interest.

## Supplementary Material

FIGURE S1 Raw images of the full x-ray films for the Western blot results reported in Figure 5 of the main text. Top Left = tyrosine hydroxylase (TH) in olfactory bulb (OB) tissue. Top Right = D2DR in OB tissue. Middle Left = ERK in OB tissue. Middle Right = TH in prefrontal cortex (PFC) tissue. Bottom Left = D2DR in PFC tissue. Bottom Left = ERK in PFC tissue. Each of the eight lanes are in the following order: 1 = WT/S, 2 = WT/M, 3 = Kv/S, 4 = Kv/M, 5 = WT/S, 6 = WT/M, 7 = Kv/S, 8 = Kv/M; where WT = wildtype, Kv = Kv1.3−/−, S = saline gavaged, M = MPH gavaged. Please see “Materials and Methods” section of the main text for antibody concentrations and Western blot procedure.
