# Supplementary figures and images for: Elevated Anxiety and Impaired Attention in Super-Smeller, Kv1.3 Knockout Mice
Source: Front Behav Neurosci. 2018 Mar 19;12:49. doi: 10.3389/fnbeh.2018.00049 (PMC5867313; doi:10.3389/fnbeh.2018.00049)

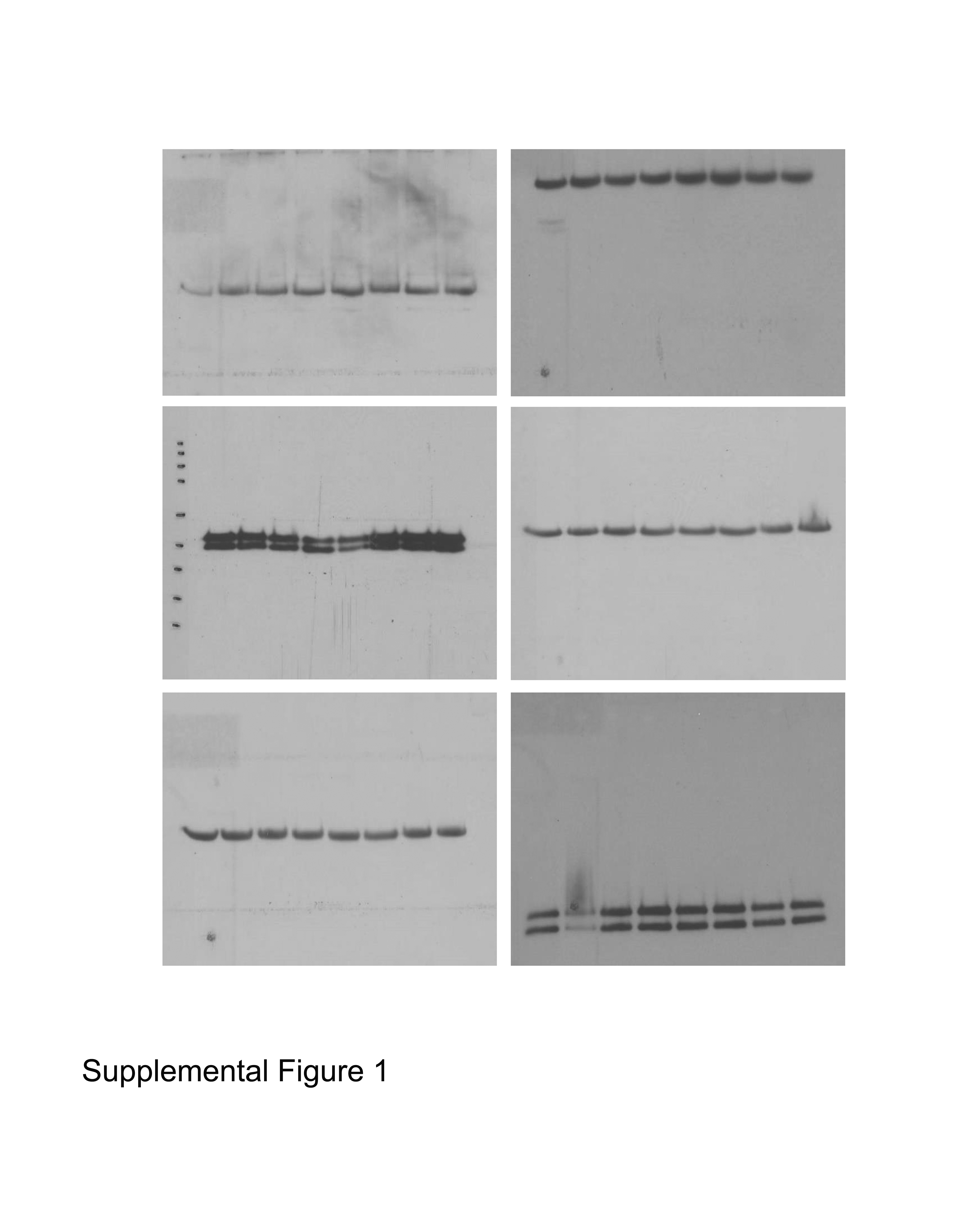

Supplement: FIGURE S1 — Raw images of the full x-ray films for the Western blot results reported in Figure 5 of the main text. Top Left = tyrosine hydroxylase (TH) in olfactory bulb (OB) tissue. Top Right = D2DR in OB tissue. Middle Left = ERK in OB tissue. Middle Right = TH in prefrontal cortex (PFC) tissue. Bottom Left = D2DR in PFC tissue. Bottom Left = ERK in PFC tissue. Each of the eight lanes are in the following order: 1 = WT/S, 2 = WT/M, 3 = Kv/S, 4 = Kv/M, 5 = WT/S, 6 = WT/M, 7 = Kv/S, 8 = Kv/M; where WT = wildtype, Kv = Kv1.3−/−, S = saline gavaged, M = MPH gavaged. Please see “Materials and Methods” section of the main text for antibody concentrations and Western blot procedure. [file Image_1.TIF]
